# HPV Genotype Distribution, Vaccine Protection Gaps, and Co-Infection Network Centrality Among 8515 Clinical Women in Wuxi, China: A Retrospective Cross-Sectional Study

**DOI:** 10.3390/vaccines14090741

**Published:** 2026-08-27

**Authors:** Baocai Ran, Han Wu, Fengjuan Zhang

**Affiliations:** 1 Department of Clinical Laboratory, Xishan People’s Hospital of Wuxi City, No. 1128 Dacheng Road, Xishan District, Wuxi 214105, China; 2 Department of Social Medicine, Graduate School of Medicine, Hirosaki University, 5 Zaifu-cho, Hirosaki 036-8562, Aomori, Japan; 3 Information Big Data Center, Affiliated Hospital of Jiangnan University, No. 200 Huihe Road, Binhu District, Wuxi 214122, China

**Keywords:** human papillomavirus, HPV genotype distribution, vaccine protection gap, co-infection network, degree centrality, hub genotype, cervical cancer, Wuxi, nonavalent vaccine, STROBE

## Abstract

**Background/Objectives**: Regional HPV genotype data for the Yangtze River Delta remain scarce, limiting evidence-based vaccine and screening policies. This study characterised the HPV genotype distribution, the vaccine protection gaps, and the co-infection network structure among clinical women in Wuxi, a prefecture-level city in the Yangtze River Delta region of Jiangsu Province, China. **Methods**: We retrospectively analysed all 8515 cervical HPV genotyping records from Xishan People’s Hospital of Wuxi City (January 2024–June 2025) using a 23-type fluorescent PCR panel classified by the International Agency for Research on Cancer (IARC) criteria. **Results**: The overall HPV prevalence was 22.83% (1944/8515; 95% CI 21.9–23.8%). The five leading types were HPV52 (4.82%), HPV58 (3.14%), HPV16 (2.96%), HPV53 (2.50%), and HPV42 (2.08%); HPV18 (0.74%) ranked below two unvaccinated carcinogens, HPV51 (1.76%, IARC Group 1) and HPV68 (1.68%, Group 2A). The age-stratified prevalence was U-shaped (χ^2^ = 123.27, *p* < 0.001), peaking at ≤20 years (52.94%; exploratory, n = 51) and ≥61 years (34.66%). Among the 1944 women with positive results, 43.0% were completely unprotected by the nonavalent vaccine, and 60.4% harboured at least one non-9vHPV type; HPV51 and HPV68 together accounted for 15.08% of the positive cases. In the co-infection network (n = 585 multiple-type infections), HPV52 achieved the highest degree centrality (281 co-infection events; normalised degree = 0.0218) and appeared in 8 of the 15 most frequent dual-type pairs, with observed/expected ratios of 6.5–14.8-fold. **Conclusions**: These findings reveal a clinically substantial vaccine protection gap and support the prioritisation of HPV51 and HPV68 in next-generation vaccine design.

## 1. Introduction

Cervical cancer kills approximately 350,000 women annually; GLOBOCAN 2022 recorded 660,000 new cases globally [1], with China contributing an estimated 150,000 new diagnoses and 55,000 deaths in the same year [2]. All invasive cervical cancers are causally attributable to persistent infection with oncogenic HPV types [3,4]; the International Agency for Research on Cancer (IARC) classifies 13 types as definite (Group 1) or probable (Group 2A) human carcinogens [5]. The WHO Global Strategy to Accelerate the Elimination of Cervical Cancer (2020) sets a 90% vaccination coverage target for adolescent girls by 2030 [6], making accurate local genotype data a prerequisite for rational policy design.

Three prophylactic vaccines are licensed in China: bivalent (HPV16/18, 2016), quadrivalent (HPV6/11/16/18, 2017), and nonavalent (HPV6/11/16/18/31/33/45/52/58, 2018) [7,8]. In China, the bivalent vaccine is given as a two-dose series (0, 6 months) for girls aged 9–14 years or a three-dose series (0, 1, 6 months) for those ≥15 years; the quadrivalent and nonavalent vaccines are each given as a three-dose series (0, 2, 6 months) and are approved for ages 9–45 years. The nonavalent formulation is estimated to prevent approximately 90% of HPV-attributable cervical cancers globally [9,10], but its real-world protection in East Asian populations (where HPV52 and HPV58 predominate rather than HPV16 and HPV18 [11,12]) remains incompletely characterised at the city level. During the study period, HPV vaccination in China was funded predominantly on a self-pay basis for the general population; Jiangsu Province, including Wuxi, piloted free bivalent-vaccine programmes for eligible schoolgirls from 2022–2023, and nationwide free bivalent vaccination for girls aged 13 was introduced only starting 10 November 2025, after our data-collection period.

Regional surveillance studies have been published for Qingdao [13], Weifang [14], Guangzhou [15], Suzhou [16], Changsha [17], Shanghai Jinshan [18], Shanghai Xuhui [19], Chengdu [20,21], and 37 other cities nationwide [22], but no large-scale, multi-type HPV epidemiological study exists for Wuxi or the broader Yangtze River Delta (a densely populated economic region spanning Shanghai and the provinces of Jiangsu, Zhejiang, and Anhui) outside Shanghai. Wuxi Xishan District had 892,400 registered residents at the end of 2024; Xishan People’s Hospital of Wuxi City is its sole tertiary-level general hospital (Grade III Class B), with an annual outpatient volume exceeding 1.14 million visits and centralised cervical HPV testing covering all inpatient and outpatient departments.

A second evidence gap concerns the age distribution. Most published series focus on women aged 25–60, leaving adolescents (≤20 years) and older women (≥61 years) poorly characterised [18,19]. Adolescents represent the primary target for pre-exposure vaccination; older women constitute a dual blind spot: they are ineligible for vaccination (upper limit 45 years in China) and are progressively disengaging from screening programmes.

A third gap concerns the co-infection network topology. Multiple-type HPV infection is associated with a substantially elevated risk of high-grade cervical intraepithelial neoplasia [23,24], and the structure of genotype co-occurrence networks may reveal biologically important interactions. Yet a formal network centrality analysis is absent from most Chinese HPV surveillance studies.

This study addressed four questions using comprehensive 23-type data from 8515 consecutive clinical women: (1) the Wuxi-specific HPV genotype prevalence profile, with type-resolved 95% confidence intervals; (2) the quantification of vaccine protection gaps across all three licensed formulations; (3) the infection characteristics at both age extremes (≤20 and ≥61 years); and (4) the co-infection network centrality of HPV52 as a candidate hub genotype, based on degree centrality metrics and observed-versus-expected co-infection ratios. A retrospective cross-sectional design was adopted because it enables the efficient use of large, centrally captured clinical genotyping records to estimate prevalence and describe co-infection patterns across a broad age range within a defined service population, providing hypothesis-generating evidence for vaccine and screening policies that can subsequently be tested in prospective cohorts.

## 2. Materials and Methods

### 2.1. Study Design and Setting

This study was conducted and reported in accordance with the STROBE guidelines for cross-sectional studies. This retrospective cross-sectional study enrolled all women who underwent cervical HPV genotyping at Xishan People’s Hospital of Wuxi City between 1 January 2024 and 30 June 2025. The Clinical Laboratory Department centralises all cervical HPV nucleic acid testing hospital-wide; results feed directly into the Laboratory Information System (LIS). Of 8516 LIS records exported, the final entry was a system-generated null row (all genotype fields negative; age and department blank) and was excluded, leaving 8515 analysable records. No a priori sample size calculation was performed; all eligible records from the study period were included to maximise precision of the 23-type genotype-specific and age-stratified prevalence estimates.

Data cleaning resolved two coding irregularities: 99 records carrying department code “1163” were verified via the Hospital Information System (HIS) as gynaecology outpatient visits and merged accordingly; 9 records coded as “internal medicine health examination” were merged with the health examination centre group (final n = 1732). As the exported dataset contained no unique patient identifiers or test dates, the analysis unit was the individual testing record. The exported LIS dataset did not include HPV vaccination history; vaccination status could therefore not be assessed or adjusted for in this analysis.

Inclusion criteria were as follows: female sex, age ≥ 16 years, and cervical exfoliated cell sample submitted for HPV genotyping as the primary clinical indication. Exclusion criteria were as follows: documented pregnancy, confirmed history of cervical surgery in HIS records, or missing or implausible age data. Eligibility was verified by HIS–LIS record linkage. This study was approved by the Institutional Review Board of Xishan People’s Hospital of Wuxi City (No. 2026-K089-01); individual informed consent was waived under Article 39 of China’s Measures for Ethical Review of Biomedical Research Involving Human Subjects given the retrospective design and use of de-identified data.

### 2.2. Specimen Collection and HPV Genotyping

Qualified gynaecologists or nurses collected cervical exfoliated cells by rotating a cervical brush five times clockwise at the external os. Specimens were immediately transferred to proprietary preservation solution (Sansure Biotech, Changsha, China), stored at 4 °C, and processed within 24 h of collection.

Genotyping used the Sansure Biotech 23-type HPV nucleic acid detection kit (Sansure Biotech Inc., Changsha, China) (fluorescent multiplexed PCR) on QuantStudio™ 5 (Thermo Fisher Scientific, Waltham, MA, USA) and SLAN-96P (Shanghai Hongshi Medical Technology, Shanghai, China) real-time PCR systems. An independently validated 23-type HPV genotyping assay using a comparable real-time PCR/minor-groove-binder-probe platform, benchmarked against the national HPV genotyping reference standard, reported a detection limit of 100 copies/µL, a coefficient of variation ≤3.18%, and 100% concordance with known genotypes in 114 clinical samples [25]. A related Sansure Biotech HPV diagnostic kit, evaluated under international reproducibility guidelines across independent European laboratories, demonstrated excellent intra-laboratory (93.8%; 95% CI 91.4–95.7; κ = 0.85) and inter-laboratory (93.4%; 95% CI 91.0–95.4; κ = 0.84) reproducibility for high-risk HPV detection, including at the individual-genotype level [26]. The panel covered 13 high-risk types (HPV16, 18, 31, 33, 35, 39, 45, 51, 52, 56, 58, 59, 68), 5 intermediate-risk types (HPV26, 53, 66, 73, 82), and 5 low-risk types (HPV6, 11, 42, 43, 81), classified per current IARC criteria [5]: HPV51 as Group 1 (definite carcinogen) and HPV68 as Group 2A (probable carcinogen). Positive and negative controls were included in every run.

### 2.3. Variable Definitions

Age groups were as follows: ≤20, 21–30, 31–40, 41–50, 51–60, and ≥61 years. Infection multiplicities were as follows: single-type (one type detected), dual-type (two types), and multiple-type infection (≥2 types).

Three vaccine coverage indices were calculated per formulation: (i) at least partial coverage (APC): ≥1 positive type falls within vaccine targets; (ii) mixed gap: ≥1 positive type falls outside vaccine targets; (iii) completely unprotected (CU): all positive types fall outside vaccine targets. APC and CU are mutually complementary (APC + CU = total positives). For bivalent and quadrivalent vaccines, CU equals mixed gap since these vaccines cover only 2 or 4 types. The nonavalent vaccine targets HPV6, 11, 16, 18, 31, 33, 45, 52, and 58.

Co-infection network metrics were as follows: degree of a genotype was defined as the total number of co-infection events in which it appeared (i.e., sum of co-detected partner types across all multiple-infected patients). $Normalised\;degree=\frac{degree}{\left(N-1\right)\times M}$, where N is the number of genotypes in the panel (23) and M is the number of multiple-infected patients (585). Expected co-infection frequency for a genotype pair (A, B) was calculated under the independence assumption as follows: $E\left(A,B\right)=P\left(A\right)\times P\left(B\right)\times N_{positive}$, where P(X) is the marginal prevalence of type X in the full cohort (n = 8515). The observed/expected (O/E) ratio quantifies enrichment above independence-expected co-occurrence.

### 2.4. Statistical Analysis

Analyses used SPSS 26.0 (IBM Corp., Armonk, NY, USA) and Python 3.11 (network metrics). HPV prevalence and 95% confidence intervals (CIs) were estimated by the normal approximation. Between-group prevalence comparisons used Pearson’s χ^2^ test, or Fisher’s exact test when any expected cell count was <5. Co-infection pairs were enumerated by pairwise combination of all detected types per patient. All tests were two-tailed; significance threshold was α = 0.05. No formal missing-data imputation was performed; records with missing or implausible age were excluded a priori (see inclusion/exclusion criteria).

## 3. Results

### 3.1. Overall Prevalence and Genotype Distribution

As the dataset lacked unique patient identifiers, the analysis unit was the individual testing record rather than the individual patient (see Section 2.1 and Section 5). Among the 8515 women (age 16–87 years; mean ± SD 43.4 ± 11.9 years), 1944 (22.83%; 95% CI 21.9–23.8%) tested positive for ≥1 HPV type. The high-risk, intermediate-risk, and low-risk HPV prevalence rates were 17.63%, 3.86%, and 5.55%, respectively. All 23 genotypes were detected. HPV52 ranked first (4.82%; 95% CI 4.36–5.27%), followed by HPV58 (3.14%), HPV16 (2.96%), HPV53 (2.50%), and HPV42 (2.08%). Crucially, HPV18 (0.74%; 95% CI 0.56–0.92%), the second antigen target in all the licensed vaccines, was less prevalent than the unvaccinated IARC Group 1 carcinogen HPV51 (1.76%) and the Group 2A carcinogen HPV68 (1.68%). The complete type-specific data with 95% CIs are presented in Table 1.

### 3.2. Vaccine Protection Gaps

Of the 1944 HPV-positive women, 57.0% (n = 1109) had at least partial nonavalent vaccine coverage (APC), while 43.0% (n = 835) were completely unprotected (CU; all detected types absent from 9vHPV), and 60.4% (n = 1174) harboured ≥1 non-9vHPV type (mixed gap). For 2vHPV and 4vHPV, the CU rate equalled the mixed-gap rate (84.0% and 79.4%, respectively), as these formulations cover only two or four types (Table 2).

Among the 14 genotypes excluded from 9vHPV, the five highest-burden types in the women with positive results were HPV53 (10.96%), HPV42 (9.10%), HPV81 (8.54%), HPV51 (7.72%), and HPV68 (7.36%). HPV51 and HPV68 together accounted for 15.08% of all the positive cases, exceeding the combined prevalence-weighted contribution of the 9vHPV-covered types HPV31 (3.66%) and HPV45 (0.98%) and underscoring a carcinogen–coverage asymmetry in the current vaccine formulations.

### 3.3. Age-Stratified Prevalence: U-Shaped Distribution

**Figure 1:**
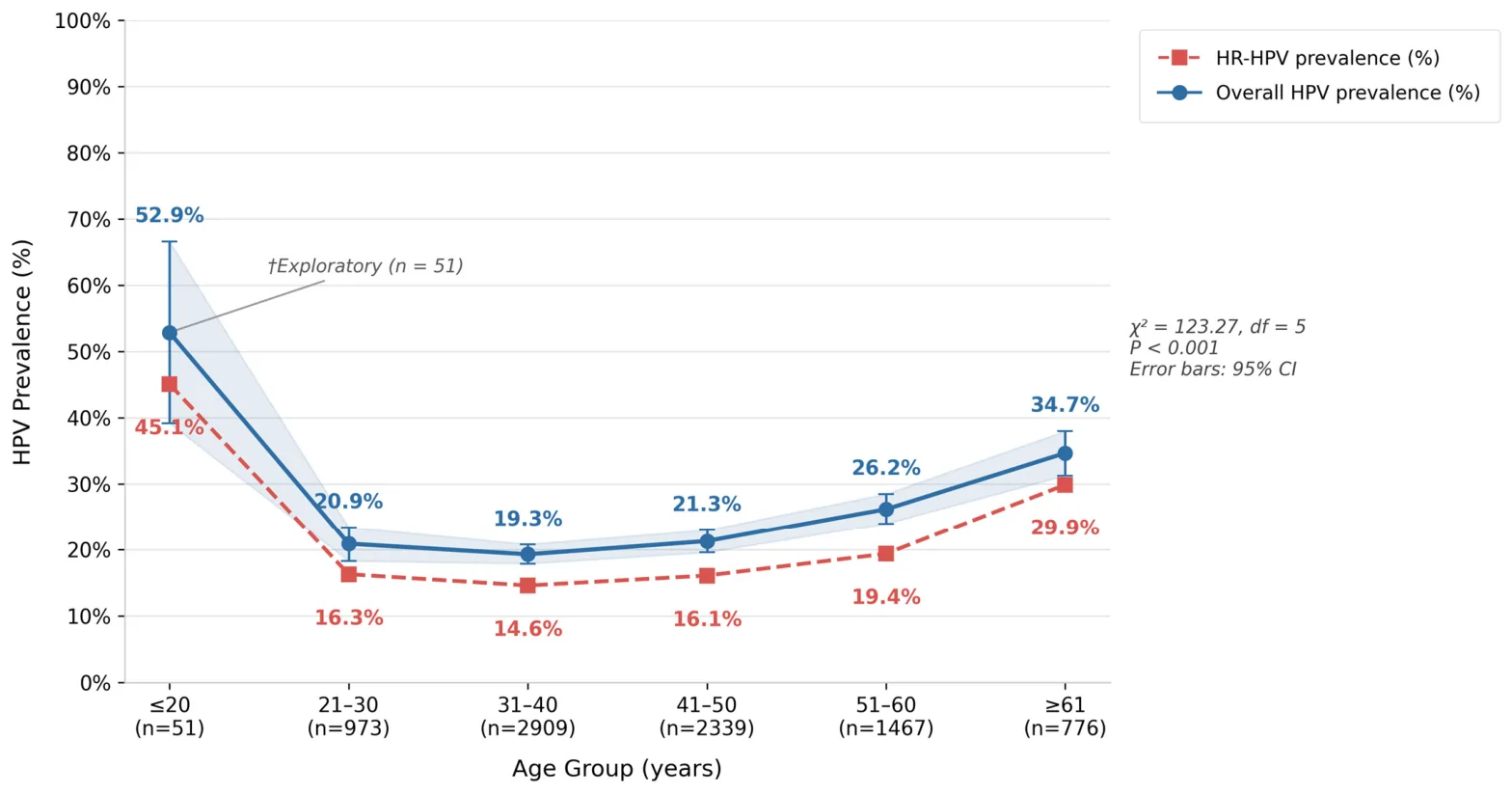
Age-stratified HPV prevalence (overall and high-risk) among 8515 clinical women in Wuxi, China (January 2024–June 2025). Blue circles with solid lines: overall HPV prevalence with 95% confidence intervals (error bars and shaded area). Red squares with dashed lines: high-risk HPV (HR-HPV) prevalence. † The ≤20-year group (n = 51; 0.60% of cohort) consists entirely of clinical attendees; results are exploratory and must not be extrapolated to the general adolescent population. χ^2^ = 123.27, df = 5, *p* < 0.001.

The prevalence differed significantly across the six age groups (χ^2^ = 123.27, df = 5, *p* < 0.001; Figure 1), tracing a U-shaped curve. It peaked in the ≤20-year group (52.94%; 95% CI 39.2–66.6%; n = 51; exploratory finding; see Table 3 footnote), fell to a nadir in the 31–40-year group (19.32%), then rose progressively, reaching 34.66% (95% CI 31.3–38.0%) in women aged ≥61 years. The high-risk HPV prevalence in the ≥61-year group was 29.90%, the highest among the non-exploratory strata. Triple-or-higher type infection was most frequent at both extremes: 13.73% (7/51) in ≤20 years and 5.28% (41/776) in ≥61 years. The nonavalent CU rate was lowest in the ≥61-year group (31.6%) versus 43.3–47.9% in the 21–60-year groups, consistent with a higher background prevalence of 9vHPV-targeted types in older women (Table 3 and Table 4).

### 3.4. Co-Infection Network Centrality of HPV52

Of the 1944 positive women, 1359 (15.96%) had a single-type infection and 585 (6.87%) had a multiple-type infection: dual, 400 (4.70%); triple, 134 (1.57%); quadruple, 30 (0.35%); quintuple, 12 (0.14%); sextuple 6 (0.07%); septuple, 1 (0.01%); and octuple, 2 (0.02%).

**Figure 2:**
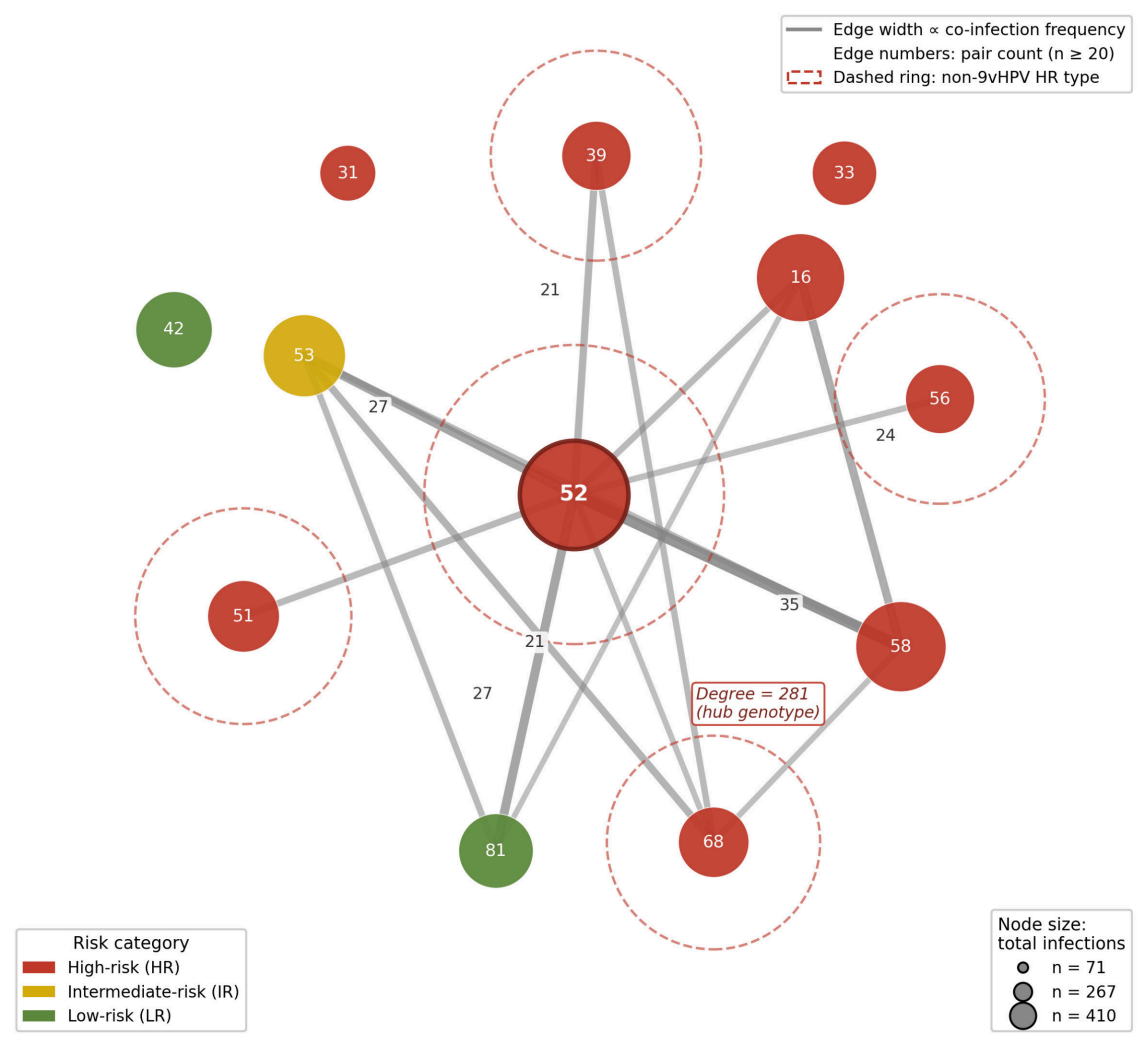
Co-infection network among 585 women with multiple-type HPV infections (Wuxi, China, 2024–2025). Node size is proportional to the total number of infections for each genotype. Edge width is proportional to co-infection frequency; numbers on edges indicate pair counts for pairs with n ≥ 20. Node colour indicates risk category: red = high-risk (HR); yellow = intermediate-risk (IR); green = low-risk (LR). Dashed red rings identify HR types absent from the nonavalent (9vHPV) vaccine. HPV52 achieved the highest degree centrality (degree = 281; normalised degree = 0.0218), consistent with a hub-genotype co-infection architecture. Co-infection pair counts for HPV52 exceeded independence-expected values by 6.5–14.8-fold (Table 6).

The network centrality analysis across the 585 multiply infected women revealed HPV52 as the highest-degree node (degree = 281 co-infection events; normalised degree = 0.0218), substantially exceeding HPV58 (degree = 212), HPV53 (207), HPV81 (181), and HPV16 (174) (Table 5; Figure 2). HPV52 co-detected with all the other 22 panel genotypes, yielding 21 distinct co-infection partner types. Among the five most frequent HPV52-anchored pairs, the observed co-infection frequencies exceeded the independence-expected values by 6.5–14.8-fold (Table 6), indicating a non-random co-occurrence enrichment. HPV52 appeared in 8 of the 15 most frequent dual-type pairs. Notably, the pairs HPV53 + HPV68 (n = 21) and HPV58 + HPV68 (n = 16) featured HPV68, a Group 2A carcinogen not covered in the current vaccines.

### 3.5. Department-Stratified Prevalence

Prevalence differed significantly across the six departments (χ^2^ = 183.0, df = 5, *p* < 0.001; Table 7). The cervical specialty clinic recorded the highest rate (33.09%; 95% CI 29.2–37.0%), 2.4-fold the health examination centre reference (13.63%; 95% CI 12.0–15.2%). A monotonic gradient was observed tracking clinical referral intensity: cervical specialty clinic (33.09%) > gynaecology OPD (26.37%) > gynaecology inpatient (16.42%) > health examination centre (13.63%). The gynaecology inpatient–health-exam-centre difference did not reach statistical significance (*p* = 0.056), limiting causal interpretation. An additional 85 cases from low-volume non-gynaecological departments were retained in the overall prevalence numerator but excluded from between-department comparisons; the six analysed departments totalled 8430 women.

### 3.6. Risk Stratification

Of 8515 women, 6571 (77.17%) were HPV-negative. Among 1944 positive women: high-risk only 1201 (14.10%); intermediate-risk only 159 (1.87%); low-risk only 260 (3.05%); high-risk + intermediate-risk 111 (1.30%); high-risk + low-risk 154 (1.81%); intermediate-risk + low-risk 24 (0.28%); all three risk tiers 35 (0.41%).

## 4. Discussion

Four findings define the clinical and public-health significance of these data. First, HPV52 emerges as a hub genotype, dominating both the overall prevalence and the co-infection network centrality, a pattern with direct implications for surveillance priorities. Second, a substantial proportion of the HPV-positive women remain unprotected by the nonavalent vaccine, with the unvaccinated carcinogens HPV51 and HPV68 constituting a disproportionate share of the residual burden relative to several vaccine-covered types. Third, the elevated prevalence observed at both age extremes identifies two prevention populations that fall outside the current vaccination and screening priorities. Fourth, the marked gradient across the clinical departments indicates that the hospital-wide prevalence figures substantially overstate the community-level burden, with the health examination centre offering the more policy-relevant baseline estimate.

HPV52 dominance and hub-genotype topology. The displacement of HPV16/18 from the top prevalence position by HPV52 is consistent across East Asian epidemiology [11,12,13,14,15,16,17,21,27,28] and has been attributed to genomic and immunological characteristics that facilitate immune persistence in East Asian host populations [29]. In our network analysis, HPV52 co-detected with all 22 other panel types and achieved a normalised degree of 0.0218, 29% above HPV58 (0.0165). The observed co-infection rates for HPV52 with its four most frequent partners were 6.5–14.8× the independence expectation, suggesting a co-occurrence enrichment that may reflect either shared exposure windows or an impaired type-specific immune clearance. This contrasts with hub-genotype analyses in other populations that implicate HPV16 as the dominant co-infection node [30]. The continued dominance of HPV52 post-9vHPV introduction warrants prospective type-replacement surveillance [14].

Vaccine protection gaps and antigen prioritisation. The 43.0% completely unprotected rate exceeds the figures reported from Suzhou (21.7%) [16], Beijing [31], and several other Chinese cities [32,33,34], reflecting both our broader 23-type panel and the referral-enriched sample. HPV51 (1.76%) and HPV68 (1.68%) together constituted 15.08% of the positive cases, individually exceeding the cohort-wide prevalence of 9vHPV-covered HPV31 (0.83%) and HPV45 (0.22%) and collectively surpassing their sum. Both meet IARC carcinogenicity criteria [5,35], and epidemiological studies have attributed 3–5% of cervical cancers globally to HPV51 and HPV68 [36]. Broader-spectrum vaccines incorporating these types are in development [37]; our local frequency and O/E co-occurrence data provide a quantitative rationale for prioritising HPV51 and HPV68 in next-generation antigen selection for the East Asian market.

U-shaped age distribution: implications for prevention policy. The trough at 31–40 years and the re-elevation at ≥61 years (HR-HPV 29.90%) replicate a pattern documented in Shanghai [18,19], Weifang [14], and Chengdu [20,21], and it mirrors the age-specific multiple-infection pattern reported nationally [38]. Immunosenescence-driven viral reactivation and impaired T-cell-mediated clearance are the most widely cited mechanisms [39,40]; the lower 9vHPV CU rate in women ≥61 (31.6% versus 43.3–47.9% in younger groups) is consistent with a higher background carriage of vaccine-targeted types accumulated over longer exposure histories. Given that Chinese recommendations cap HPV vaccination at age 45 and that elderly women progressively disengage from screening, routine high-risk HPV screening continuation beyond age 65 and a colposcopy referral for HPV-positive women aged ≥61 are warranted, consistent with the current Chinese cervical cancer screening guidelines [41]. The ≤20-year finding (52.94%; n = 51) is exploratory; all the cases were clinical attendees with an inherent selection bias, and the wide 95% CI (39.2–66.6%) reflects the small sample. Nevertheless, a similarly elevated rate in this stratum has been reported in Shanghai [18,19] and Chengdu [20], and the HPV16 prevalence of 15.69%, the highest in all the age strata, is biologically plausible given rapid high-risk exposure shortly after sexual debut [42]. These data support a clinician-initiated HPV risk assessment for sexually active patients below age 25 in clinical settings, without implying a need to revise the national screening age thresholds.

Department gradient and external validity. The 2.4-fold prevalence gradient directly tracking the clinical referral intensity confirms that the overall hospital-level HPV rates are strongly confounded by the case mix. The health examination centre prevalence (13.63%) closely approximates the community-level estimates from the Suzhou (10.2%) [16] and Chengdu health-check cohorts (~14%) [21], suggesting that the underlying community HPV burden in Wuxi is not markedly elevated relative to adjacent cities and that the 22.83% overall figure reflects a referral enrichment rather than an intrinsically higher population prevalence. This distinction is crucial for policy translation: local cervical cancer prevention strategies should be calibrated to the community baseline (approximately 13–14%), not the referral-enriched clinical rate.

## 5. Limitations

This single-centre retrospective cross-sectional study has six limitations. First, generalisation to the broader Wuxi population requires caution. Second, the retrospective data preclude any adjustment for key confounders including vaccination history, number of sexual partners, contraceptive use, and smoking status. The absence of vaccination status data is a particular limitation given that this study period (2024–2025) coincides with expanding 9vHPV uptake in China; we cannot determine whether the 43.0% CU rate would differ between vaccinated and unvaccinated subgroups. Individual-level HPV vaccination records were not available for this cohort. However, the national surveillance data indicate that HPV vaccination coverage among adult Chinese women remains very low: 3.2% among women aged 46–50 and 4.6% among those aged 9–17 as of 2022 [43]. A 2025 meta-analysis estimated the overall coverage among mainland Chinese women at only 9.5%, despite 70.6% stating willingness [44]. HPV vaccination is not part of China’s National Immunization Program, and free catch-up programs for adult women remain limited to select pilot regions as of the study period, not including Wuxi. Given this context, we consider it unlikely that vaccination status fully accounts for the observed genotype distribution, though a partial contribution cannot be excluded; prospective studies linking vaccination records with genotyping data are needed to resolve this. Third, the ≤20-year group (n = 51, 0.60% of cohort) consists entirely of clinical attendees; the prevalence is exploratory. Fourth, the cross-sectional design precludes any inference on infection persistence, temporal sequence, or causal direction in the co-infection pairs. Fifth, the exported dataset lacked unique patient identifiers and test dates, so the analysis unit was the individual testing record rather than the individual patient; a small number of women who submitted repeat samples during the study period were therefore counted more than once, which may have marginally influenced the prevalence estimates. Sixth, the LIS diagnostic field completion was 42.3%, preventing a systematic HPV–cytology linkage; the integration of TCT and histopathology results, alongside HPV genotyping, would substantially strengthen the clinical interpretation of these epidemiological findings [45].

## 6. Conclusions

This 23-type retrospective cross-sectional study of 8515 consecutive clinical women in Wuxi establishes four actionable conclusions. HPV52 is the dominant high-risk genotype across all the age strata, achieves the highest co-infection network degree centrality (degree = 281; normalised degree = 0.0218), and displays a 6.5–14.8-fold enriched co-occurrence with its top partners, consistent with hub-genotype behaviour. The nonavalent vaccine leaves 43.0–60.4% of the women with positive results incompletely protected; the IARC carcinogens HPV51 (Group 1) and HPV68 (Group 2A) constitute the principal unmet need, each exceeding the prevalence of 9vHPV-covered HPV31 and HPV45. A U-shaped age–prevalence distribution, with high-risk HPV rates of 29.90% in women ≥61 years and exploratory rates of 45.10% in women ≤20 years, reveals two currently underserved populations at the extremes of the age spectrum. The department-level stratification shows that the health examination centre baseline (13.63%) approximates the community-level prevalence, while the 22.83% overall rate reflects a case-mix enrichment from referral patients. These data support HPV51 and HPV68 as priority antigens for next-generation vaccine design and call for sustained cervical cancer screening coverage across the full clinically active age spectrum.

## Figures and Tables

**Table 1:** Distribution of 23 HPV genotypes ranked by prevalence (denominator n = 8515; 95% CI by normal approximation).

HPV Type	Risk Category	n	Prevalence (%)	95% CI (%)	9vHPV	Notes
HPV52	High-risk	410	4.82	4.36–5.27	No	
HPV58	High-risk	267	3.14	2.77–3.51	Yes	9v covered
HPV16	High-risk	252	2.96	2.60–3.32	Yes	2v/4v/9v covered
HPV53	Inter-risk	213	2.50	2.17–2.83	No	IARC Group 2B
HPV42	Low-risk	177	2.08	1.78–2.38	No	
HPV81	Low-risk	166	1.95	1.66–2.24	No	
HPV51 ★	High-risk	150	1.76	1.48–2.04	No	IARC Group 1; absent from all vaccines
HPV68 ★	High-risk	143	1.68	1.41–1.95	No	IARC Group 2A; absent from all vaccines
HPV39	High-risk	136	1.60	1.33–1.86	No	
HPV56	High-risk	135	1.59	1.32–1.85	No	
HPV59	High-risk	113	1.33	1.08–1.57	No	
HPV33	High-risk	111	1.30	1.06–1.54	Yes	9v covered
HPV66	Inter-risk	90	1.06	0.84–1.27	No	
HPV6	Low-risk	76	0.89	0.69–1.09	Yes	4v/9v covered
HPV43	Low-risk	73	0.86	0.66–1.05	No	
HPV31	High-risk	71	0.83	0.64–1.03	Yes	9v covered
HPV18	High-risk	63	0.74	0.56–0.92	Yes	2v/4v/9v covered
HPV35	High-risk	60	0.70	0.53–0.88	No	
HPV11	Low-risk	32	0.38	0.25–0.51	Yes	4v/9v covered
HPV82	Inter-risk	24	0.28	0.17–0.39	No	
HPV45	High-risk	19	0.22	0.12–0.32	Yes	9v covered
HPV73	Inter-risk	13	0.15	0.07–0.24	No	
HPV26	Inter-risk	6	0.07	0.01–0.13	No	

Inter-risk: intermediate-risk. ★ HPV51: IARC Group 1 (definite carcinogen); HPV68: IARC Group 2A (probable carcinogen), both absent from all currently licensed HPV vaccines. 9vHPV: nonavalent vaccine covering HPV6/11/16/18/31/33/45/52/58; 4vHPV: quadrivalent; 2vHPV: bivalent.

**Table 2:** Vaccine protection-gap analysis across three licensed HPV formulations (denominator: 1944 HPV-positive women).

Vaccine	Target Types (n)	APC [n (%)]	Mixed Gap [n (%)]	CU [n (%)]	China Approval Year
2vHPV (bivalent)	HPV16, 18 (2)	311 (16.0)	1633 (84.0)	1633 (84.0)	2016
4vHPV (quadrivalent)	HPV6, 11, 16, 18 (4)	400 (20.6)	1544 (79.4)	1544 (79.4)	2017
9vHPV (nonavalent)	HPV6, 11, 16, 18, 31, 33, 45, 52, 58 (9)	1109 (57.0)	1174 (60.4)	835 (43.0)	2018

APC: at least partial coverage (≥1 positive type within vaccine targets). CU: completely unprotected (all positive types outside vaccine targets). Mixed gap: ≥1 positive type outside vaccine targets. For 2vHPV and 4vHPV, CU = mixed gap because all non-target types constitute a complete coverage absence in these narrow-spectrum formulations. 9vHPV: nonavalent; 4vHPV: quadrivalent; 2vHPV: bivalent.

**Table 3:** Age-stratified HPV prevalence, high-risk infection, and multiple-type infection distribution.

Age (Years)	n	HPV Prevalence [n (%), 95% CI]	HR-HPV [n (%)]	Single [n (%)]	Dual [n (%)]	≥Triple [n (%)]	9vHPV CU (%)
≤20 †	51	27 (52.94), 39.2–66.6%	23 (45.10)	12 (23.53)	8 (15.69)	7 (13.73)	18.5
21–30	973	203 (20.86), 18.3–23.4%	159 (16.34)	125 (12.85)	48 (4.93)	30 (3.08)	43.3
31–40	2909	562 (19.32), 17.9–20.8%	426 (14.64)	437 (15.02)	92 (3.16)	33 (1.13)	47.9
41–50	2339	498 (21.29), 19.6–23.0%	377 (16.12)	377 (16.12) ‡	89 (3.81)	32 (1.37)	44.0
51–60	1467	385 (26.24), 24.0–28.5%	284 (19.36)	255 (17.38)	88 (6.00)	42 (2.86)	43.9
≥61	776	269 (34.66), 31.3–38.0%	232 (29.90)	153 (19.72)	75 (9.66)	41 (5.28)	31.6
Total	8515	1944 (22.83), 21.9–23.8%	1501 (17.63)	1359 (15.96)	400 (4.70)	185 (2.17)	43.0

95% CI by normal approximation. HR-HPV: high-risk HPV. 9vHPV CU: completely unprotected rate (all positive types absent from 9vHPV coverage). ≥Triple: triple through octuple infection. † ≤20-year group: n = 51 (0.60% of cohort), all clinical attendees; high 95% CI width reflects small n; results are exploratory and must not be extrapolated to the general adolescent population. ‡ In the 41–50-year group, the count for HR-HPV positives (377) coincidentally equals single-type infections (377); these are independent measures (single-type infections include 110 non-HR cases; HR-positives include 110 multiple-type women).

**Table 4:** Top three HPV genotypes by age group (prevalence within age-group denominator; high-risk types only).

Age (Years)	Rank 1 Genotype (%)	Rank 2 (%)	Rank 3 (%)	Notable Finding	n
≤20 †	HPV52 (17.65)	HPV16 (15.69)	HPV18 (9.80)	Highest HPV16 prevalence across strata	51
21–30	HPV52 (5.55)	HPV16 (2.88)	HPV58 (2.36)	—	973
31–40	HPV52 (3.47)	HPV58 (2.68)	HPV16 (2.17)	—	2909
41–50	HPV52 (3.98)	HPV58 (3.25)	HPV16 (2.95)	—	2339
51–60	HPV52 (5.39)	HPV16 (2.93)	HPV58 (2.86)	—	1467
≥61	HPV52 (9.54)	HPV58 (5.54)	HPV16 (5.28)	Rising prevalence with age	776

HPV52 ranked first in all six age groups. † Exploratory, n = 51.

**Table 5:** Co-infection network degree centrality for the six highest-degree genotypes (multiple-type infection group, n = 585 women; full 23-type panel).

Genotype	Degree (Co-Infection Events)	Normalised Degree	Direct Co-Infection Partners (n)	Observed/Expected Ratio (Top 5 Pairs)
HPV52	281	0.0218	21 (all other types)	6.5–14.8×
HPV58	212	0.0165	—	—
HPV53	207	0.0161	—	—
HPV81	181	0.0141	—	—
HPV16	174	0.0135	—	—
HPV68	156	0.0121	—	—

Degree: total number of co-infection events in which a genotype appeared across all multiple-infected patients. Normalised degree = degree/[(N − 1) × M], where N = 23 genotypes and M = 585 multiple-infected patients. Observed/expected ratios shown for HPV52’s five most frequent co-infection pairs; expected values computed under independence assumption (see Methods 2.3). “—”: O/E not computed for genotypes other than HPV52 in this table.

**Table 6:** The 15 most frequent dual-type co-infection pairs with observed/expected ratios (multiple-type infection group, n = 585).

Rank	Co-Infection Pair	Observed (n)	Expected (n) *	O/E Ratio	HPV52 Involved
1	HPV52 + HPV58	35	2.9	11.9×	Yes
2	HPV52 + HPV53	27	2.3	11.5×	Yes
3	HPV52 + HPV81	27	1.8	14.8×	Yes
4	HPV16 + HPV58	24	—	—	No
5	HPV53 + HPV68	21	—	—	No
6	HPV39 + HPV52	21	1.5	14.0×	Yes
7	HPV51 + HPV52	19	—	—	Yes
8	HPV53 + HPV81	18	—	—	No
9	HPV16 + HPV52	18	2.8	6.5×	Yes
10	HPV53 + HPV58	18	—	—	No
11	HPV39 + HPV68	18	—	—	No
12	HPV52 + HPV56	17	—	—	Yes
13	HPV52 + HPV68	16	—	—	Yes
14	HPV58 + HPV68	16	—	—	No
15	HPV16 + HPV81	15	—	—	No

* Expected co-infection count under independence: E(A,B) = P(A) × P(B) × N_positive, where P(X) is the marginal prevalence of type X in the full cohort (n = 8515) and N_positive = 1944. O/E ratios shown only for HPV52-involving pairs with n ≥ 18. “—”: O/E not computed for pairs with complex multi-way co-infection contexts or insufficient n.

**Table 7:** HPV prevalence by clinical department (Pearson χ^2^ test, reference: health examination centre).

Department	Tested (n)	Positive (n)	Prevalence (%)	95% CI (%)	χ^2^ vs. Health Exam Centre
Cervical Specialty Clinic	553	183	33.09	29.2–37.0	*p* < 0.001
Gynaecology OPD	5066	1336	26.37	25.2–27.6	*p* < 0.001
Gynaecology (Inpatient)	962	158	16.42	14.1–18.8	*p* = 0.056
Health Exam Centre	1732	236	13.63	12.0–15.2	Reference
Preventive Health Clinic	85	14	16.47	8.6–24.4	*p* = 0.560
Menopause Clinic	32	2	6.25	0–14.7	*p* = 0.343

Gynaecology OPD (n = 5066) includes 99 records originally coded as department “1163” (verified as gynaecology OPD by HIS). Health Exam Centre (n = 1732) includes 9 records coded as “internal medicine health examination”. Menopause Clinic and Preventive Health Clinic: small cell counts; *p*-values are indicative only and should not be used for between-clinic comparisons. 95% CI: normal approximation. Pairwise comparisons versus the health examination centre reference were not corrected for multiple testing; results should be interpreted descriptively rather than as confirmatory hypothesis tests.

## Data Availability

Data are available from the corresponding author on reasonable request, subject to patient privacy protections.

## Abbreviations

The following abbreviations are used in this manuscript:

2vHPV	Bivalent HPV vaccine
4vHPV	Quadrivalent HPV vaccine
9vHPV	Nonavalent HPV vaccine
APC	At least partial coverage
CI	Confidence interval
CU	Completely unprotected
HIS	Hospital Information System
HPV	Human papillomavirus
HR-HPV	High-risk human papillomavirus
IARC	International Agency for Research on Cancer
IRB	Institutional Review Board
LIS	Laboratory Information System
O/E	Observed/expected ratio
OPD	Outpatient department
PCR	Polymerase chain reaction
SD	Standard deviation
STROBE	Strengthening the Reporting of Observational Studies in Epidemiology
TCT	Thinprep cytologic test
WHO	World Health Organization
